# 123 Green/grey spaces - What is the best urban setting to enhance physical activity? Preliminary findings of the Health Benefits of Outdoor Physical Activity (HOPA) study

**DOI:** 10.1093/eurpub/ckae114.072

**Published:** 2024-09-26

**Authors:** Thayse Natacha Gomes, Adam Sullivan, Sara Suikkanen, Triin Rääsk, Saima Kuu, Ilkka Väänänen, Alan Donnelly

**Affiliations:** Department of Physical Education and Sport Sciences, Health Research Institute, Physical Activity for Health Research Cluster, University of Limerick, Limerick, Ireland; Department of Physical Education and Sport Sciences, Health Research Institute, Physical Activity for Health Research Cluster, University of Limerick, Limerick, Ireland; Physical Activity and Functional Capacity Research Group, Faculty of Health Care and Social Services, LAB University of Applied Sciences, Lahti, Finland; Institute of Natural and Health Sciences, Tallinna University, Tallinn, Estonia; Institute of Natural and Health Sciences, Tallinna University, Tallinn, Estonia; Physical Activity and Functional Capacity Research Group, Faculty of Health Care and Social Services, LAB University of Applied Sciences, Lahti, Finland; Department of Physical Education and Sport Sciences, Health Research Institute, Physical Activity for Health Research Cluster, University of Limerick, Limerick, Ireland

## Abstract

**Purpose:**

Strategies aimed at increasing physical activity (PA) must be feasible and easy for participants to adhere to. Outdoor public spaces emerge as relevant venues due to their easy accessibility and lack of cost. However, it is not clear what kind of urban spaces (“nature-based” or “road/sidewalk-based”) contribute the most to increasing PA. This study seeks to investigate the effect of an outdoor intervention on enhancing PA levels and reducing sedentary time (SED) among inactive adults.

**Methods:**

HOPA was a multi-centre parallel-group randomised control trial (ISRCTN64480977), carried out in three European cities (Lahti, Limerick, Tallinn). The sample comprised 101 adults, self-reported as inactive at baseline (IPAQ), aged between 25-65 years. Participants were instructed to engage in outdoor PA (walking/running) at least three times/week, at least 30 minutes/session, over eight weeks. They were randomly allocated into two groups, based on the type of outdoor spaces where they would perform their activities: “nature-based” and “road/sidewalk-based” spaces. PA (light PA, LPA; moderate-to-vigorous PA, MVPA) and SED were measured at baseline and during week-8 of the intervention, over 9-consecutive days, using activPAL accelerometers. Between- and within-group differences were analysed using General Linear Model, adopting a significance level of 95%.

**Results:**

There were no significant between-groups differences. A significant time-effect was observed for total weekly LPA (ρ = 0.014;η^2^=0.06) and MVPA (ρ < 0.001;η^2^=0.126), but not for weekly average SED (ρ = 0.117). Specifically, LPA decreased from 511.1min/week to 489.1min/week in “nature-based” group, and from 539.3min/week to 498.2min/week in “road/sidewalk-based” group. Conversely, MVPA increased from 219.5min/week to 262min/week in “nature-based” group, and from 227.6min/week to 269.6min/week in “road/sidewalk-based” group. No significant group-time effect was observed, indicating that both “nature-based” and “road/sidewalk-based” groups provide changes in PA.

**Conclusions:**

Outdoor spaces can be relevant venues for promoting PA engagement and increasing MVPA. Further analysis can provide insights into the relationship between environmental quality and PA, but these preliminary findings suggest that both “nature-based” and road/sidewalk-based” spaces have shown to be relevant to getting people active. Urban cities must develop strategic plans aimed at designing urban spaces that are conducive to PA, promoting its practice, that would positively impact population health.

**Funding:**

EU-2020 (DOI:10.3030/869764).

